# Novel “double-strut” fibula ankle arthrodesis for large tumor-related bone defect of distal tibia

**DOI:** 10.1186/s12891-019-2742-6

**Published:** 2019-08-09

**Authors:** Zhiqing Zhao, Taiqiang Yan, Xiaodong Tang, Wei Guo, Rongli Yang, Shun Tang

**Affiliations:** Musculoskeletal Tumor Center, Peking University People’s Hospital, No.11 Xizhimen South Street, Xicheng District, Beijing, 100044 China

**Keywords:** Distal tibia, Bone tumors, Limb salvage, Ankle arthrodesis

## Abstract

**Background:**

Reconstruction for large bone defect of distal tibia after wide resection of tumor is difficult, and the best option remains controversial. This study presents a novel “double-strut” fibula ankle arthrodesis for this issue.

**Methods:**

Nine patients with malignant or aggressive tumors of distal tibia underwent novel “double-strut” fibula ankle arthrodesis after wide tumor resection were retrospectively reviewed. We assessed the bone union time, complications and oncology outcome clinically and radiographically. The Musculoskeletal Tumor Society (MSTS) score and the Foot and Ankle Outcome Score (FAOS) were used to evaluate the functional outcome.

**Results:**

The average followup period was 53 ± 46 months. There was no deep infection or graft fracture observed in this series. Internal fixation loosening was found in one case. In these patients, eight achieved union at both proximal and distal junctions, while one achieved union only distally. The mean union time of the proximal junctions and distal junctions was 10.5 ± 1.6 months and 8.7 ± 2.3 months, respectively. The mean postoperative MSTS score was 83% ± 8%. The subscales of FAOS indicating the most problem was Sport and Recreation Function with a mean score of 18 ± 11. At the final follow-up, one of them (1/9, 11%) experienced local recurrence in soft tissue and received another resection surgery, and four (4/9, 44%) patients developed lung metastases.

**Conclusions:**

For large bone defect of distal tibia, this novel “double-strut” fibula reconstruction can be a viable alternative, which is capable of achieving durable ankle fusion and functional salvaged limb with low rate of complications.

**Electronic supplementary material:**

The online version of this article (10.1186/s12891-019-2742-6) contains supplementary material, which is available to authorized users.

## Background

The distal tibia is an uncommon site of occurrence for primitive bone tumors, there are no large series covering this issue [1–3]. In the past decades, below-knee (B-K) ablation was the standard treatment for malignant bone tumors and for local recurrences of aggressive bone tumors of distal tibia [4]. Nowadays, advanced chemotherapy and surgical techniques made limb salvage possible, and previous studies have proved that limb salvage can achieve acceptable functional outcome and survival rates compared with ablative technique [5–13]. Various reconstruction options have been reported in literature, including massive allograft, recycled tumor-bearing bone, vascularized or non-vascularized autografts, prosthetic replacement or bone transport [7, 8, 12, 14–21]. However, to our knowledge, no consensus has been reached concerning the gold standard treatment since each technique can be accompanied with certain disadvantages. The postoperative complications were reported to range from 4 to 92% [4, 5, 8, 11, 12, 14, 15, 20–25]. And the functional result assessed by Musculoskeletal Tumor Society (MSTS) score ranged from 20 to 100% [5, 9–12, 14, 20, 24, 26, 27].

In 1987, a study conducted by Jupiter et al. reported the use of a divided fibular shaft with the peroneal and medullary vessels to the proximal strut and the peroneal vessels to the distal strut for treating large defect in the femoral shaft [28]. This technique named “double barrel” or “double-strut” fibula reconstruction increases the volume of bone to a given length of defect by two-fold while maintaining the free fibular transfer without increasing the microvascular anastomosis, and it was used in many other studies treating large defects in long bones [18, 29, 30].

**Fig. 1:**
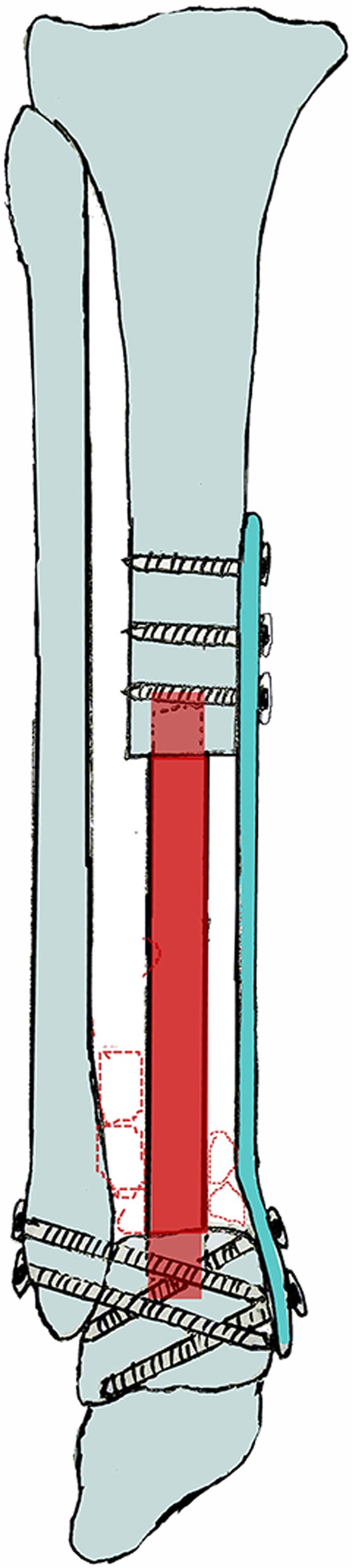
Schematic of novel “double-strut” fibular reconstruction—the non-vascularized fibular shaft harvested from contralateral limb is inserted to tibia canal and talus, served as intercalary spacer, which parallels to the ipsilateral fibula. The iliac crest serves as adjuvant graft can be placed between two fibulas to enhance ankle bone union. The talus, ipsilateral fibula, and plate make up a triangle of stability

In this study, we described a novel “double-strut” fibula reconstruction—the non-vascularized fibula transfer was inserted to remaining tibia canal and talus, which parallels to the ipsilateral fibula—to restore limb continuity (Fig. 1). Nine patients received this new technique after tumor resection of distal tibia and achieved satisfactory results. Our study aimed to provide a viable alternative for reconstruction of large bone defect of distal tibia.

## Methods

This study was approved by the Ethics Committee of Peking University People’s Hospital and informed consent was waived due to the retrospective nature of this study. We retrospectively reviewed the data of all patients with primary malignant or aggressive tumors of the distal tibia treated in our center from September 2003 to October 2017. Nine of them were reconstructed by novel “double-strut” fibula and internal fixator, with ankle arthrodesis. Table 1 showed the indications and contraindications of this procedure.

**Table 1:** The indications and contraindications to double-strut fibula ankle arthrodesis surgery

Indications	Contraindications
1. Aggressive or malignant bone tumor of distal tibia, intracompartmental or extracompartmental	1. Tumor involving the crucial neurovascular tissue
2. Possibility of wide resection obtaining negative surgical margins	2. Intra-articular extension of the tumor
	3. Tumor with ipsilateral fibula involvement
	4. Tumor with large soft tissue mass hardly obtain a safe wide margin

There were five males and 4 females with a mean age of 26 ± 9 years. Six patients were diagnosed with osteosarcoma (OS) and one with malignant giant cell tumor (MGCT) histopathologically confirmed. Two patients with giant cell tumor of distal tibia were previously treated with tumor curettage and bone grafting, and local relapse developed later. Further needle biopsy confirmed recurrent giant cell tumor (RGCT) of distal tibia (Table 2). Among these 9 cases, seven with OS or MGCT were stage IIB and two with RGCT were stage 3 according to the Enneking classification adopted by the Musculoskeletal Tumor Society [31].

**Table 2:** Clinical details of the patients

No.	Age/Sex	Diagnosis	Stage	RL cm	Bone union time (month)		Complication	MSTS %	FU month	Oncology
					Proximal	Distal				
1	17/M	OS	IIB	20	12	9	Met	87	151	NED
2	20/M	OS	IIB	15	12	9	Met	83	48	DOD
3	22/F	OS	IIB	15	9	9		83	84	NED
4	22/M	MGCT	IIB	7	9	9	Met	87	24	DOD
5	21/M	OS	IIB	14	12	12		90	84	NED
6	45/M	OS	IIB	11	12	6		87	39	NED
7	33/F	OS	IIB	13	9	6	LR/Met	87	22	NED
8	22/F	RGCT	3	8	9	6		67	10	NED
9	28/F	RGCT	3	11	/	12	Screw loosening	73	14	NED

*M* Male, *F* Female, *RL* Resection length, *MSTS* Musculoskeletal Tumor Society Score, *FU* Followup time, *OS* Osteosarcoma, *MGCT* Malignant giant cell tumor, *RGCT* Recurrent giant cell tumor, *Met* Lung metastasis, *LR* Local recurrence, *NED* No evidence of disease, *DOD* Died of disease

X-ray, MRI, CT scan and radionuclide scan were used for initially evaluation. Five patients with conventional osteosarcoma received two cycles of neoadjuvant chemotherapy. One (patient 6) with well-differentiated intraosseous osteosarcoma and other three patients did not receive any chemotherapy. Re-evaluation was taken at the end of the preoperative chemotherapy. Surgery was performed 2 or 3 weeks after the neoadjuvant chemotherapy.

### Surgical technique

The procedure includes four main steps: 1. Harvesting non-vascularized fibular graft from the contralateral limb, 2. En-bloc resection of the tumor, 3. Reconstruction of the bone defect, and 4. Ankle arthrodesis and fixation by plate osteosynthesis.

#### Harvesting the non-vascularized autogenous fibular graft

The non-vascularized fibula, which was at least 2 cm longer than the resected tibia detected by MRI, was harvested from the contralateral limb. In order to protect the common peroneal nerve, enough length (at least 5 cm) from the tip of fibular head was left. Meanwhile, distal fibular longer than 8–10 cm was preserved as well to maintain the stability of lateral malleolus, otherwise, the talus and lateral malleolus were fused by screws.

#### Wide en bloc excision of distal tibia tumor

**Fig. 2:**
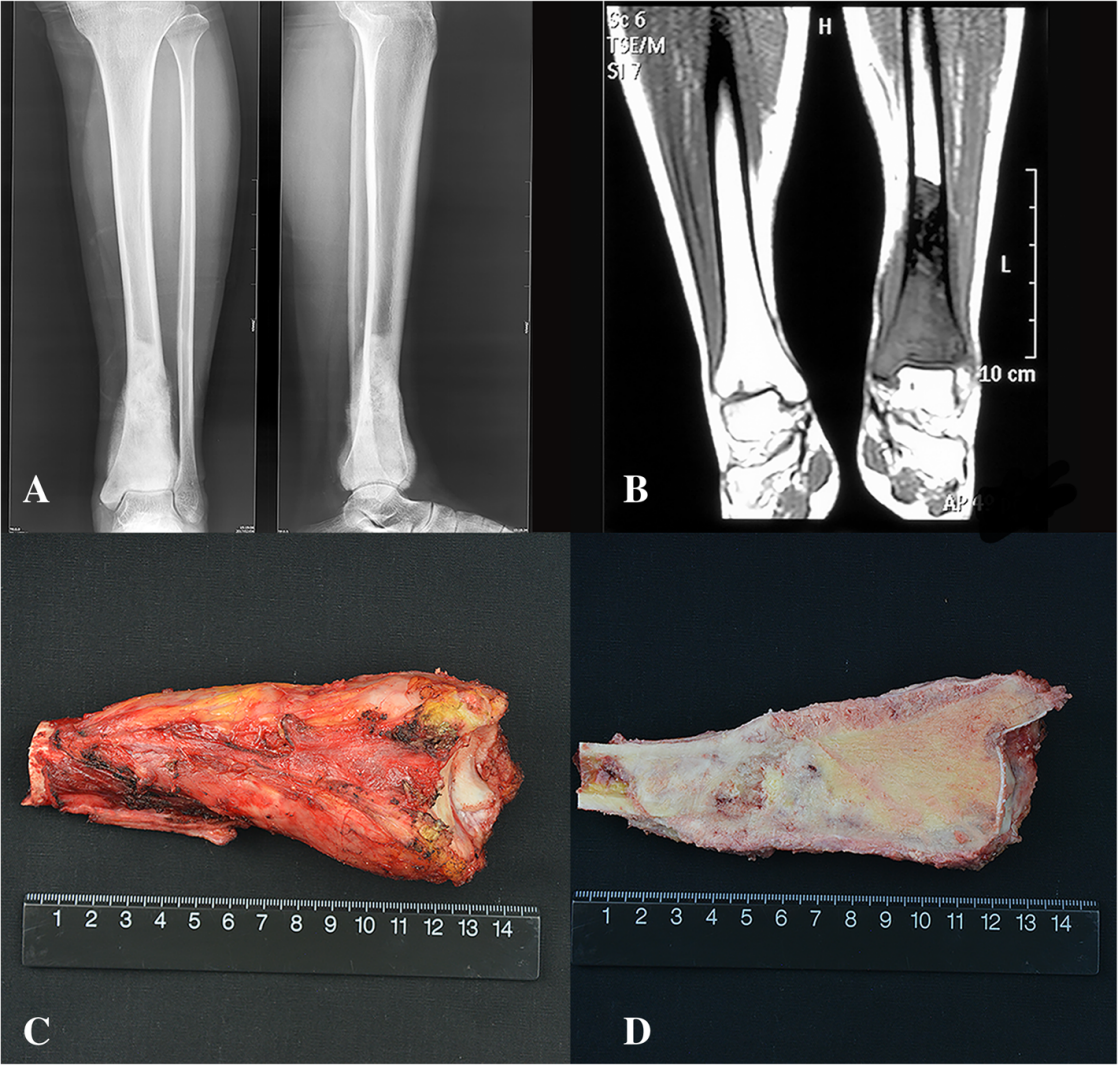
**a** Preoperative X-ray film showing osteogenic osteosarcoma of distal tibia associated with periosteal reaction (Patient 7). **b** MRI coronal T1 weighted image showing osteoblastic bone destruction of distal tibia with confined soft tissue mass. **c** Tibia osteotomy was performed at least 2 cm above the upper margin of the tumor as determined by MRI. **d** The excised specimen showing the intraosseous lesion and satisfactory surgical margins

The distal tibia was approached through an anterolateral incision. Previous biopsy tract was incorporated into the incision and completely excised with the specimen. An intraarticular distal tibia resection was done with meticulous dissection carried out to preserve a wide protective margin of tissue. The level of tibial resection was based on the proximal extent of the tumor as determined by MRI, osteotomy was performed at least 2 cm above the upper margin of the tumor (Fig. 2). The average resection length of tibia was 12.7 ± 4.0 cm. Bone marrow from the remaining proximal tibia was sent to the laboratory for pathological evaluation.

#### Reconstruction of the bone defect

**Fig. 3:**
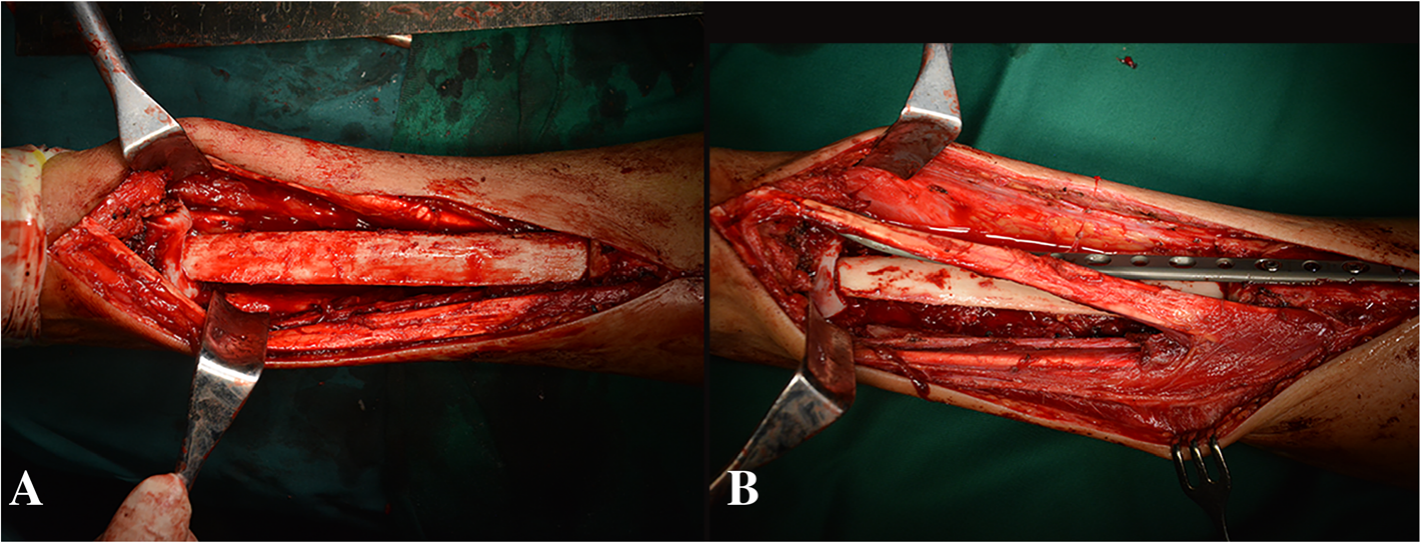
Intraoperative image showing that (**a**) one end of the fibula transfer was inserted into the proximal residual tibia medulla, and the other end was inserted into the notch of native talus. **b** A long compression plate and screws fixation were used to bridge the gap between the remaining tibia and talus

After tumor resection, a notch (1 cm) was then made in the upper dome of the talus to accept the bone graft. One end of the aforesaid non-vascularized fibula was placed into the proximal residual tibia medulla after widening it with a reamer, and the other end was inserted into the notch of native talus (Fig. 3).

#### Ankle arthrodesis and fixation

**Fig. 4:**
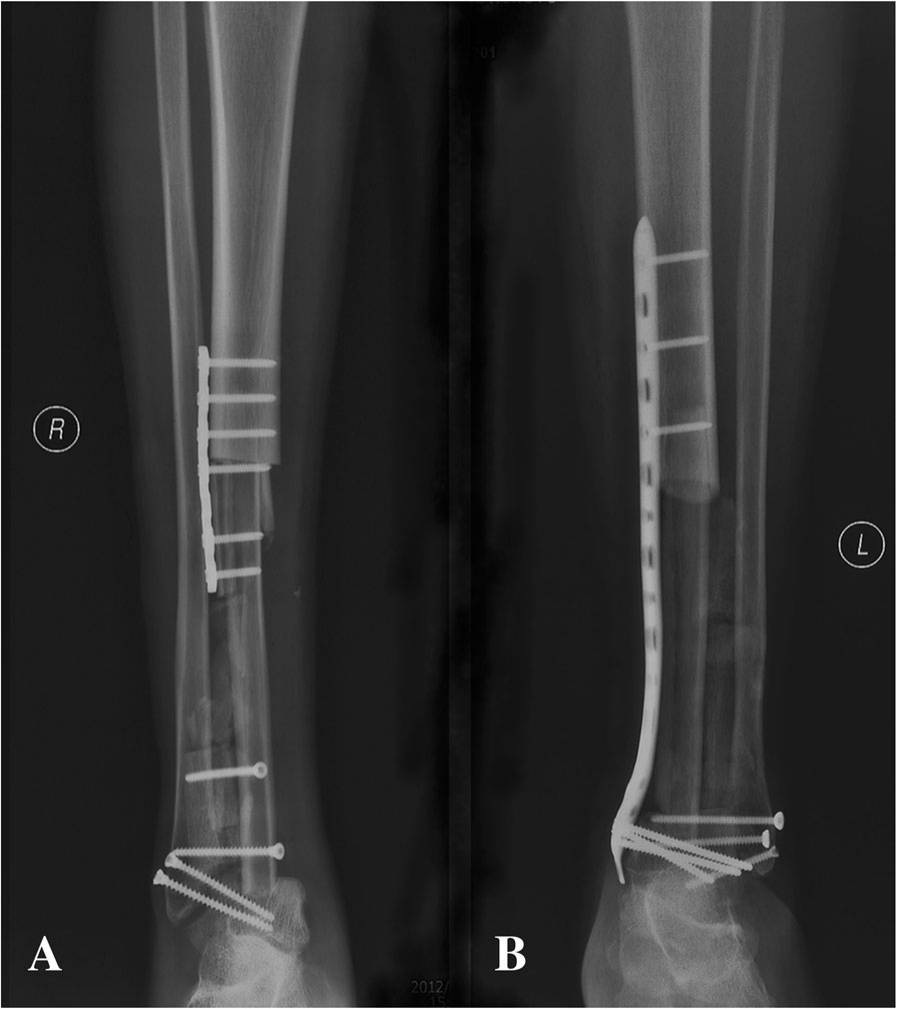
The postoperative X ray films showing that (**a**) the proximal graft-host junction was fixed by plate and distal junction by intercross screws, or **b** Long compression plate and screws fixation

The proximal graft-host junction was fixed by plate and distal junction by intercross screws in four patients, while long compression plate and screws fixation were used to bridge the gap between the remaining tibia and talus (Fig. 4) in the other 5 patients. Care was taken to adjust the ankle in neutral dorsiflexion with 5° to 10° of the valgus and 10° external rotation. In order to further enhance ankle stability, the talus and ipsilateral fibula were fused together by screws after cartilage surface removal. Supplemental autologous or allogeneic bone chips were added in the space between two fibulas.

#### Rehabilitation

All patients were kept non-weightbearing for 3 months, then progressed to partial weightbearing wearing a brace thereafter, and were allowed to bear full weight depending on radiologically confirmed bone union. Signs of bony union were evaluated using serial sets of plain X-ray films. We defined union as uninterrupted external bony borders between the graft and host with obscured or absent osteotomy lines at both junctions, and union of the arthrodesis when trabeculation was seen across the site of the arthrodesis [32].

### Followup

Clinical outcomes were assessed by review of clinic notes, supplemented by phone questionnaires. Function was evaluated using the functional rating system adopted by the Musculoskeletal Tumor Society (MSTS) in 1993 [33]. Simultaneously, we investigated patient-reported functional outcome using the Foot and Ankle Outcome Score (FAOS) [34]. The rating system was designed to evaluate symptoms and functional limitations related to the foot and ankle, consisting of 42 items assessing five subscales: Pain; Other Symptoms like stiffness, swelling, and range of motion; Activities of Daily Living; Sport and Recreational Activities; and Foot and Ankle-related Quality of Life. Sum up the total score of each subscale and then transformed to a zero to 100, which 100 indicates no problems and 0 indicates extreme problems [34]. The end-point for analysis was the last followup visit or death.

### Statistical analysis

The statistical analysis was carried out by using SPSS software version 22.0 (IBM Corp., Armonk, New York, USA). Distributions of quantitative variables were expressed as mean ± standard deviation (SD). The comparison of bone healing time between proximal junctions and distal ones was performed using independent t-test. A *P*-value ≤0.05 was considered to be statistical significance.

## Results

**Fig. 5:**
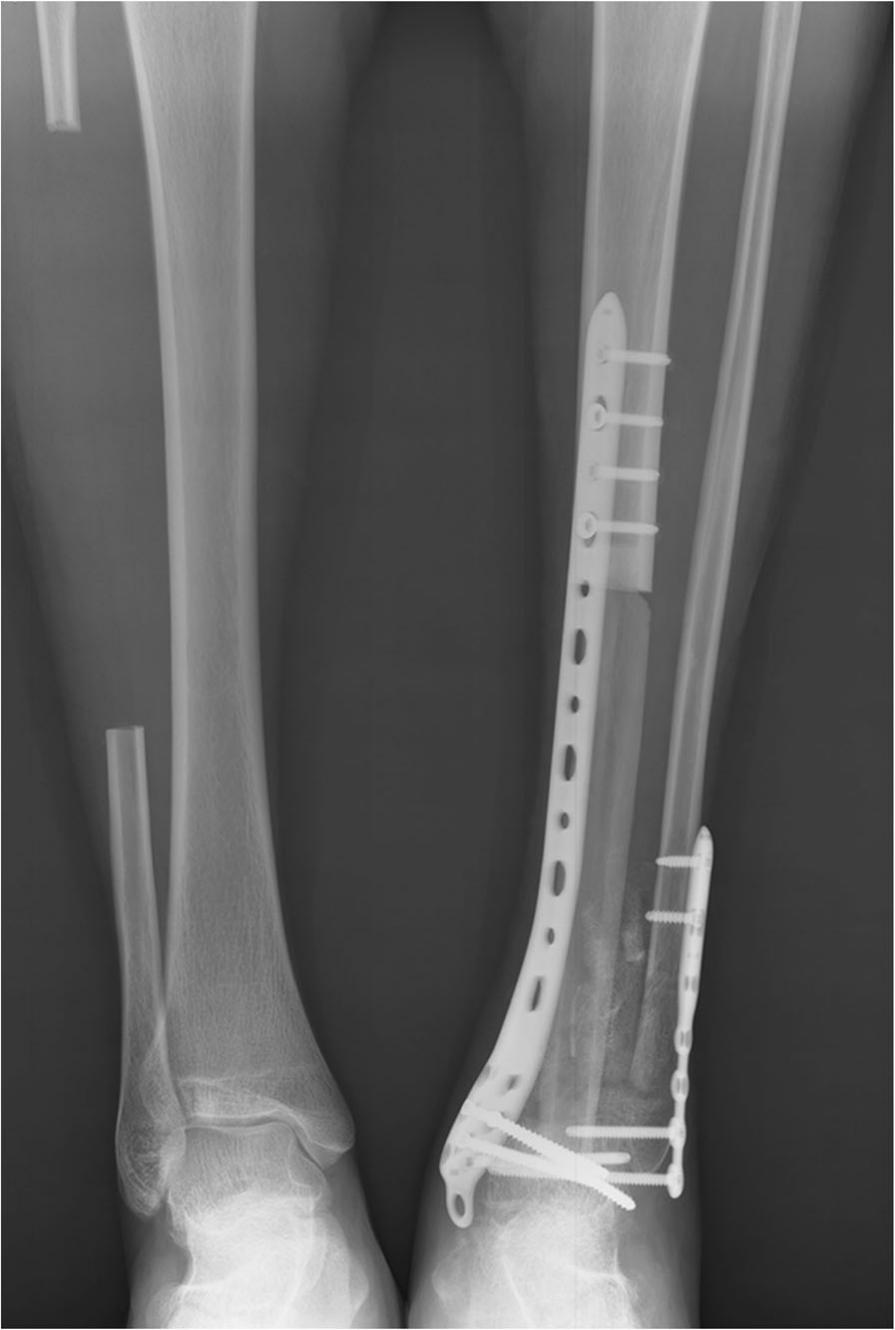
One intraoperative complication was observed in patient 7 that the distal ipsilateral fibula was fractured during reconstruction, therefore, additional plate osteosynthesis was applied

The mean duration for the whole procedures was 3.3 ± 0.8 h. The average blood loss was 417 ± 229 ml. One intraoperative complication was observed in patient 7 that the distal ipsilateral fibula was fractured during reconstruction, therefore, additional plate osteosynthesis was applied (Fig. 5).

**Fig. 6:**
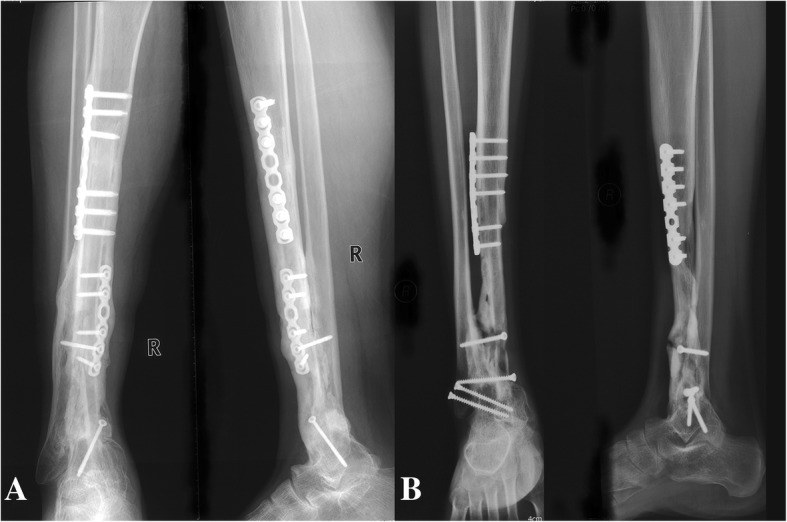
**a** The radiograph obtained at 30 months after surgery showing the fusion of the graft and host bone (Patient 1). **b** Postoperative 24 months radiograph showing successful fusion in both proximal and distal junctions (Patient 5)

**Fig. 7:**
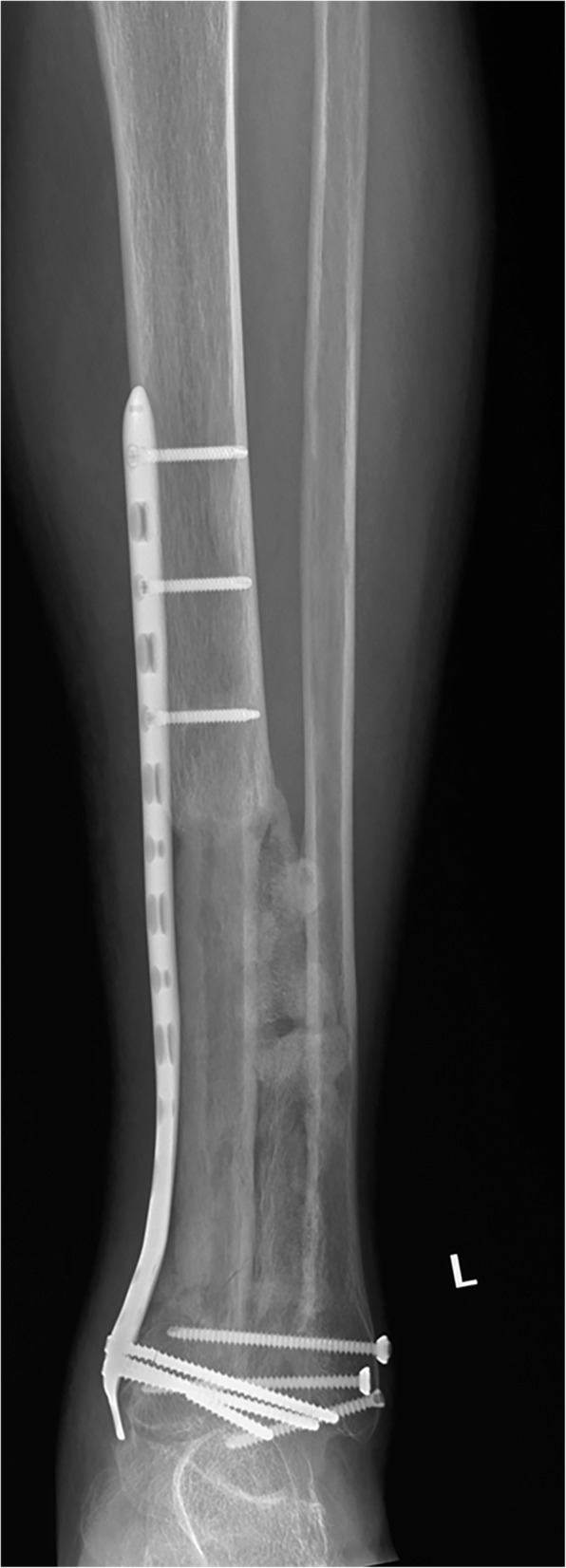
Plain radiograph taken 22 months after surgery showing that the fibula transfer is becoming hypertrophy under weight-bearing stimulation (Patient 6)

**Fig. 8:**
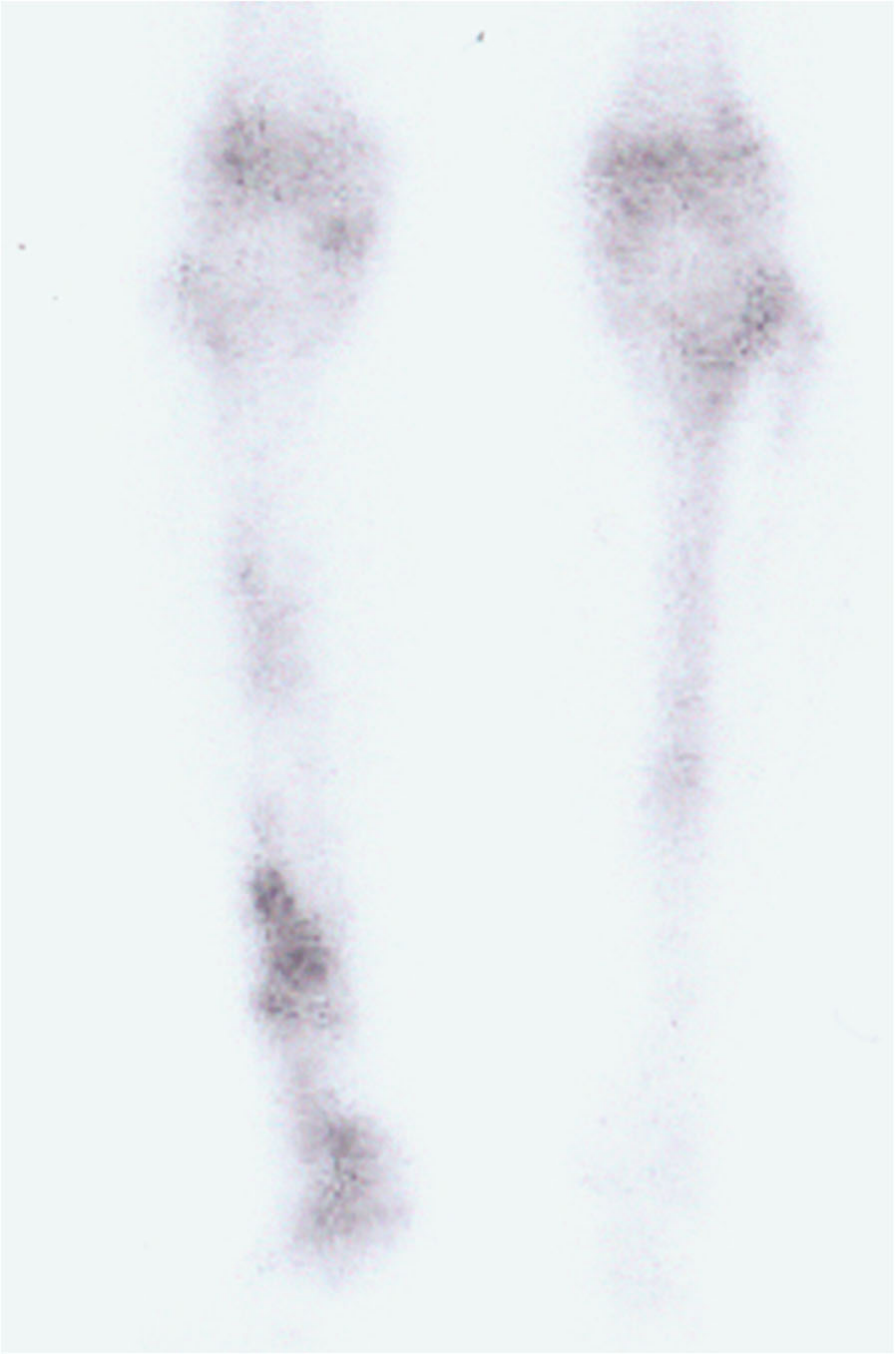
The bone scan image taken 30 months after surgery indicating a greater radionucleotide uptake at whole fibula graft (Patient 1)

No patient was lost to followup. The average followup period was 53 ± 46 months. One internal fixation loosening occurred and was managed by re-osteosynthesis. Graft healing went on to demonstrate radiographically. The latest X films shown 8 cases achieved successful bone union at both proximal and distal junctions (Fig. 6) except one only achieved union distally. The mean union time of the proximal junctions was 10.5 ± 1.6 months and that of the distal junctions was 8.7 ± 2.3 months. The average union time of the proximal junctions was similar with that of the distal junctions (*P =* 0.083). The fibula transfer was seen to be hypertrophy when the limb was mechanically loaded (Fig. 7). Furthermore, a greater radionucleotide uptake at whole fibula graft could be observed from bone scan image (Fig. 8), revealing that vascularity was achieved. The postoperative functional MSTS score ranged from 67 to 90%, averaged 83 ± 8%. Regarding the patient-reported outcome evaluated by FAOS, the mean scores of Pain, Other Symptoms, Activities of Daily Living, Sport and Recreational Activities, and Foot and Ankle-related Quality of Life were 93 ± 9, 71 ± 11, 83 ± 21, 18 ± 11, and 64 ± 15, respectively (Table 3). Eight patients could walk on level ground without pain, return to their former occupations and some entertainment activities, and the other one patient (patient 9) still need a crutch to walk (Additional files 1 and 2 show the postoperative function of patients 6 and 7 respectively).

**Table 3:** The Foot and Ankle Outcome Score of patients

No.	Pain	Other Symptoms	Activities of Daily Living	Sport and Recreational Activities	Quality of Life
1	100	75	93	25	63
2	94	75	70	20	44
3	94	71	90	20	69
4	100	79	94	10	63
5	100	82	96	40	75
6	100	82	91	5	69
7	97	57	91	25	81
8	81	68	93	10	75
9	75	50	32	10	37


**Additional file 1:** **Video 1.** The video shows the limb functional of patient 6 at 30 months after surgery. (MP4 11344 kb)



**Additional file 2:** **Video 2.** The video shows the functional outcome of patient 7 at 6 months postoperatively. (MP4 17199 kb)


At the final followup, one of them experienced local relapse in soft tissue and she received another resection surgery. Four patients (4/9, 44%) developed lung metastases. Among the four patients, one (patient 1) with solitary lesion and underwent metastasis removal by thoracoscopic surgery and achieved free disease thereafter, one is still alive with lung metastatic disease, 2 (patients 2 and 4) died of systematic disease.

## Discussion

In this study, nine patients underwent novel “double-strut” fibula reconstruction after tumor resection of distal tibia, and achieved satisfactory result. We presented this novel technique of bone grafting for distal tibial defect, aiming to serve as an alternative option for the orthopaedic surgeon.

The distal tibia is undoubtedly a critical site for the treatment of malignant lesions. B-K amputation is still a treatment option, especially for those patients whose tumor with poor response to chemotherapy, involving the crucial neurovascular bundle, or limb salvage failure. A systematic review concluded that limb salvage can provide similar function of the limb as compared to amputated limbs (77.1% vs 70.9%, *P* = 0.055) for distal tibia tumor [13]. The reported recovery (walking with no crutches) time from B-K amputation was 61.1 ± 11.4 days in patients with war-related amputations and 80.9 ± 8.1 days in patients with vascular disease-related amputations [35], which was less than the time for limb salvage (about 1 year). However, most patients still request to preserve the limb due to cosmetic and psychological demands. Limb salvage surgery for this location is a unique, perplexing, and problematic task for the reason of the limited amount of soft tissue coverage and the complicated biomechanical factors [15, 36, 37]. Nevertheless, limb salvage is preferred for those patients in whom the preoperative imaging was suggestive that satisfactory surgical margins could be achieved. We have conducted a systematic review and reported 86.1% of patients with distal tibia tumor received limb salvage procedures and achieved acceptable outcome [13].

It is well known that prosthetic replacement is rather uncommon and less successful compared with the other major joint replacements (knee, hip, and shoulder) due to lack of muscle coverage in this anatomic region that will complicate reconstruction with metallic implants, and burdened with long-term complications including deep infection, loosening, and ankle instability [6, 7, 10]. On the other hand, the ankle joint motion is relatively dispensable; ankle arthrodesis is an old and widely accepted procedure in orthopedic surgery and has almost no real functional disadvantages [4, 5, 8, 14, 17, 23, 24].

Reconstruction with ankle arthrodesis using bone grafts is still a good and safe technique, has been the method of choice by most orthopaedists. The bone grafts can be autografts (taken from other anatomic regions of the same individual) and tumor-bearing bone graft after devitalization, or can be allografts. Besides, the allograft, duly shaped in gutter-like fashion, can be used in combination with the vascularized or non-vascularized fibula as described by Capanna et al. [19]. In spite of massive allograft can achieve good outcome, it is still associated with a significant set of complications such as high risk of infection, and nonunion, requiring a long period of non-weightbearing postoperatively to avoid graft fracture. Besides, allograft is not available in some countries due to that bone banking requires substantial time, energy, and money [38]. Furthermore, the osteoallograft maybe not applicable or necessary because it is very bulky for the dimensions of this site due to the limited soft tissue coverage.

The fibular strut graft is the most common autograft which is easy to obtain and results in minimal donor-site morbidity. It can also fit perfectly inside the medullary canal of the tibia. Fibula transfer is able to become hypertrophy under weight-bearing stimulation [14, 39, 40], it encourages us to try fibular reconstruction. Our novel “double-strut” fibula ankle arthrodesis technique is similar to the reported methods. Bishop’s study reported the use of vascularized fibula grafts harvested from ipsilateral limb for distal tibial bone defect longer than 4 cm in four cases, ankle arthrodesis were all successfully achieved [25]. Shalaby et al. reported 6 patients of osteosarcoma in distal tibia. Non-vascularized fibular graft in 3 patients and vascularized fibular graft in 3 patients and the tibiotalar arthrodesis was fixed by external fixator. 83% of patients had successful bone fusion except 1 local recurrence [8]. In Zhang’s study, a technique that dual ipsilateral vascularized fibular graft and ankle arthrodesis was reported [26]. The proximal osteotomized free vascularized fibula harvested from ipsilateral limb was placed on the medial side of the talus distally and medial side of the remaining tibia proximally, the talus was fused together with the adjacent double fibula by screws, then the external fixator was applied. All 5 cases achieved sound fusion with a mean time of 7 months.

Unlike these methods mentioned above, in our study, all the fibular grafts were harvested from contralateral limb and none of them was vascularized. Meanwhile, we preserved the ipsilateral fibula and fused with talus, it can serve as an ancillary structure for weight bearing. Initial fixation of the arthrodesis was obtained by plate osteosynthesis, bridging the residual tibia and the talus. In our opinion, this method is more adequate than massive allograft because soft tissue coverage is easier, in addition, autologous or allogeneic bone chips can be inserted to the surgical bed to enhance the chance of bone healing.

We have found low rate of complication and satisfactory functional outcome of patients treated by “double-strut” fibula ankle arthrodesis in our previous study [27]. In this current study, 8 out 9 patients achieved bone union at both the proximal and distal junctions. The average functional MSTS score was 83%, which was comparable to previous reports [5, 8, 11, 12, 24]. The subscales of FAOS indicating the most problem was Sport and Recreation Function with a mean score of 18 ± 11. After all, arthrodesis is a function-limiting procedure, although with some restriction in sports such as running, jumping, twisting and kneeling, the patients with solid ankle fusion in this study could walk on level ground without pain, function very well during the activities of normal daily living, and return to their former occupations and some entertainment activities.

The advantages of this novel “double-strut” fibula technique are as follows: (1) Compared with osteoallograft or combination of an allograft with the vascularized fibula flap (Capanna’s method), the fibula transfer is more suitable in volume that can be surrounded by abundant soft tissue, making skin closure easier and low rate of deep infection. (2) Internal fixation can provide initial stability for earlier weight bearing, fibula graft improved the osteogenesis and provided structure support as well, as time goes on, union and progressive hypertrophy of the fibula transfer will further strengthen ankle stability and the capacity for weight bearing. (3) Additionally, micro-vascular anastomosis is not required, simplifying operative procedures.

Vascularized fibular grafts was widely used in many studies [4, 5, 8, 11, 19, 25, 32, 41]. However, in this study, the mean resection length of the tibia was 12.7 ± 4.0 cm, the vascularized fibular graft was not used, and all the non-vascularized fibular got bone union or showed sign of union at both ends and gradually became hypertrophy under weight bearing.

In our study, the surgical technique was only performed in selected patients whose tumors did not involve the ipsilateral fibula. Otherwise, we would choose other surgical options, such as allograft or recycled pasteurized tumor-bearing autograft. As to patients whose tumor with large soft tissue mass hardly obtain a safe margin, B-K amputation should be considered still.

Some limitations in this study we must acknowledge. Firstly, it is a retrospective study with a small number of cases. After all, patients with primary tumors affecting the distal tibia are uncommon. Secondly, it may not be possible to judge the true incidence of postoperative complications due to our relative short follow up time, longer term followup was needed to justify this. Finally, it would strengthen our finding if we had performed gait and mechanical analyses to explored biomechanical effects of ankle arthrodesis.

## Conclusions

For large bone defect of distal tibia, this novel “double-strut” fibula reconstruction can be a viable alternative, which is capable of achieving durable ankle fusion and functional salvaged limb with low rate of complications.

## Data Availability

The datasets used and/or analyzed during the current study are available from the corresponding author on reasonable request.

## Abbreviations

B-K: Below-knee
FAOS: Foot and Ankle Outcome Score
MGCT: Malignant giant cell tumor
MSTS: Musculoskeletal Tumor Society
OS: Osteosarcoma
RGCT: Recurrent giant cell tumor
